# Dental caries and periodontal status in children and adolescents with type 1 diabetes mellitus: an umbrella review

**DOI:** 10.1007/s40368-026-01224-9

**Published:** 2026-06-10

**Authors:** Athanasia Pappa, Panagiotis Koromantzos, Kyriaki Seremidi, Aris Liakos, Adriani Nikolakopoulou

**Affiliations:** 1 Department of Paediatric Dentistry, National and Kapodistrian University of Athens, Athens, Greece; 2 Department of Periodontology, National and Kapodistrian University of Athens, Athens, Greece; 3 Clinical Research & Evidence-Based Medicine Unit, Second Medical Department, Aristotle University of Thessaloniki, Thessaloniki, Greece; 4 Laboratory of Hygiene, Social-Preventive Medicine & Medical Statistics, School of Medicine, Aristotle University of Thessaloniki, Thessaloniki, Greece

**Keywords:** Dental caries, Periodontitis, Gingivitis, Diabetes mellitus, Child, Adolescent

## Abstract

**Purpose:**

To summarise and evaluate the evidence from systematic reviews on the oral health of children and adolescents with type 1 diabetes mellitus (T1DM).

**Methods:**

A literature search was conducted in four databases for systematic reviews, with or without meta-analysis, reporting on dental caries experience and/or periodontal health of patients up to 19 years of age with T1DM, compared to healthy controls. Study selection and data extraction were performed independently by two reviewers and findings were synthesised descriptively. Overlap of primary studies was assessed using the Corrected Covered Area and methodological quality using the ROBIS tool.

**Results:**

Eleven systematic reviews were included, from a total of 3437 records identified. Each review included a range of initial studies (2–36), identifying a total of 82 primary studies (very high overlap). Most were cross-sectional with sample sizes ranging between 174 and 2519 in T1DM and 217– 2490 in healthy controls. Five reviews were of high risk of bias, with the remaining being either of low (*n* = 3) or unclear (*n* = 3) risk. Caries experience did not differ significantly in primary dentition, whereas in the permanent dentition, some reviews reported an increased experience prevalence, particularly in those with poorer glycaemic control. Greater gingival inflammation and periodontal tissue destruction were observed in children and adolescents with T1DM.

**Conclusion:**

Children and adolescents with T1DM may be at greater risk of periodontal disease, while evidence on dental caries remains inconsistent. Methodological heterogeneity across studies emphasises the need for well-designed longitudinal research and high-quality systematic reviews with standardised outcome measures.

**Supplementary Information:**

The online version contains supplementary material available at https://doi.org/10.1007/s40368-026-01224-9.

## Introduction

Type 1 diabetes mellitus (T1DM) is a chronic autoimmune condition characterised by the destruction of insulin-producing β-cells in the pancreas, leading to absolute insulin deficiency and persistent hyperglycaemia (ADA 2025). Onset most commonly occurs during childhood or adolescence but can also develop later in life (Buzzetti et al. 2017). T1DM represents a growing global health concern, affecting approximately 9.5 million individuals worldwide, 1.9 million (19%) of whom are younger than 20 years of age, with over 500,000 new cases diagnosed annually (Ogle et al. 2025).

In children and adolescents, T1DM usually presents abruptly, with diabetic ketoacidosis being the initial manifestation (ADA 2025). Chronic hyperglycaemia is associated with multiple systemic complications, particularly microvascular alterations leading to conditions such as diabetic nephropathy, retinopathy and neuropathy (Giri et al. 2018). Amongst systemic manifestations, oral findings are also commonly seen mainly due to high blood glucose levels that alter salivary production and secretion. Apart from increased prevalence of common dental diseases (dental caries and periodontal disease), alterations in oral health have also been reported, including increased susceptibility to infections, mucosal changes, taste disturbances, burning mouth symptoms and delayed wound healing (Rohani 2019).

Overall, findings regarding dental caries and T1DM remain relatively unclear. Several systematic reviews (SRs) report greater dental caries experience amongst patients with T1DM (Liu et al. 2021; Triebl et al. 2024), whereas others find no statistically significant differences between groups (Ismail et al. 2015; Nassar et al. 2024). Of note, glycaemic control appears to be a key modifying factor; poor metabolic regulation, as indicated by higher HbA1c levels, has been associated with increased caries lesion formation, higher counts of acidogenic bacteria and more pronounced periodontal inflammation (Rapone et al. 2020; Ta et al. 2025).

In contrast, studies suggest that young patients with T1DM usually have a less favourable periodontal risk profile compared to healthy controls (Jensen et al. 2021). The underlying mechanisms are both biological and behavioural. Immune and inflammatory dysregulation, changes in salivary composition and flow rate, and shifts in the oral microbiota can create a microenvironment more susceptible to disease, whilst dietary patterns and oral hygiene may further contribute to this risk (Ferizi et al. 2018; Ta et al. 2025; Triebl et al. 2024).

Due to the growing number of SRs addressing oral health outcomes in children and adolescents with T1DM, an umbrella review was conducted, in order to critically evaluate the consistency and robustness of the available evidence (Belbasis et al. 2022). Thus, the aim of the present study was to summarise and evaluate the existing evidence from SRs and meta-analyses (MAs) regarding dental caries experience and periodontal disease indices in children and adolescents with T1DM.

## Materials and methods

### Protocol and registration

This umbrella review was performed in accordance with the Preferred Reporting Items for overviews of reviews (PRIOR) statement (Gates et al. 2022) and the protocol was prospectively registered in the PROSPERO international prospective register of systematic reviews (Registration number: CRD420251103557).

### Eligibility criteria

The selection criteria were defined using the PECO framework for SRs with or without MAs on children and adolescents (P) diagnosed with T1DM (E) compared to age and gender-matched systemically healthy controls (C) for prevalence of common oral diseases (O).

Included were systematic reviews with primary cross-sectional, case series, case–control or cohort studies reporting on:

- patients up to the age of 19 years
- diagnosed with T1DM according to specific diagnostic criteria
- compared to systemically healthy children and adolescents
- prevalence of dental caries [DMFT/dmft, DT, MT (Klein et al. 1938)] or periodontal disease [PI (Plaque Index) (Löe and Silness 1963), GI (Gingival Index) (Silness and Löe 1964), Calculus Index (Greene and Vermillion 1964), BoP (Bleeding on Probing) (Ainamo and Bay 1975), OHI (Oral Hygiene Index) (Greene and Vermillion 1964) and other periodontal indices].

Narrative reviews, scoping reviews, literature reviews and systematic reviews focusing on adult or mixed populations without separating paediatric data, assessing only T1DM patients without a healthy control group and evaluating other common dental diseases were excluded from the umbrella review.

### Search strategy

A comprehensive search was conducted using both free-text words and Medical Subject Headings (MeSH Terms) in the following databases: MEDLINE/PubMed, Cochrane Database of Systematic Reviews, Scopus, Web of Science (Appendix A). In addition, abstracts from conferences of relevant scientific societies, including the European Academy of Paediatric Dentistry (EAPD), the American Academy of Paediatric Dentistry (AAPD), the European Association for the Study of Diabetes (EASD) and the International Society for Paediatric and Adolescent Diabetes (ISPAD), were reviewed. The reference lists of the included SRs were also screened manually to identify any additional relevant publications.

### Study selection

Two authors (AP, PK) independently conducted the database search. After removing duplicate records, the titles and abstracts of all retrieved records were screened. In cases where eligibility could not be determined based on the title or abstract alone, the full text of the article was reviewed. Reasons for exclusion at the full-text stage were recorded. Any discrepancies between reviewers were resolved through discussion, or by consulting a third reviewer (KS).

### Data extraction and management

Data were extracted using a customised form that included the following variables: author; year of publication; study type; number of included studies; tool used for methodological quality assessment; quantitative synthesis; main findings. Where available, quantitative data reported in the MAs were also extracted, including effect measures [mean difference (MD), standardized mean difference (SMD)], 95% confidence intervals (CI) and heterogeneity estimates (I^2^). Two reviewers (AP, PK) carried out data extraction independently, and any disagreements were resolved through discussion, or if needed by consulting a third reviewer (KS).

### Data synthesis

Descriptive analysis of the results was performed and presented in summary tables that included the main SR characteristics and findings. Data were grouped by oral health outcomes, including dental caries and periodontal parameters. Additionally, mean values were calculated based on the reported study-level data included in each SR, without sample size weighting.

### Overlapping of SRs

To evaluate the extent of overlap among the included SRs, a citation matrix was created, in which each row represented a primary study and each column an SR. Each primary study appearing in an SR was indicated with a check mark, which allowed for easy visualisation of shared studies across SRs (Bougioukas et al. 2021). Based on this matrix, the degree of overlap was calculated by the Corrected Covered Area (CCA) (Pieper et al. 2014), using the formula: CCA = (N-r)/(r*c-r) (where N is the total number of included publications—including double counting –, r is the number of unique primary studies—rows in the citation matrix— and c is the number of SRs—columns in the matrix). CCA values below 5% indicate slight, 6–10% moderate, 11–15% high and ≥ 15% very high overlap (Pieper et al. 2014). In addition, pairwise CCA was calculated to assess the extent of overlap between each pair of SRs (Pieper et al. 2014).

### Quality assessment

The methodological quality of eligible SRs and MAs was assessed independently by two reviewers using the Risk of Bias in Systematic Reviews (ROBIS) tool (Whiting et al. 2016)—ROBIS evaluates risk of bias across the following main domains: definition of eligibility criteria, identification and selection of studies, methods of data extraction and appraisal, synthesis and interpretation of results. It also assesses the overall confidence of the SR. Any disagreements between reviewers were resolved through discussion.

## Results

### Study selection

**Fig. 1:**
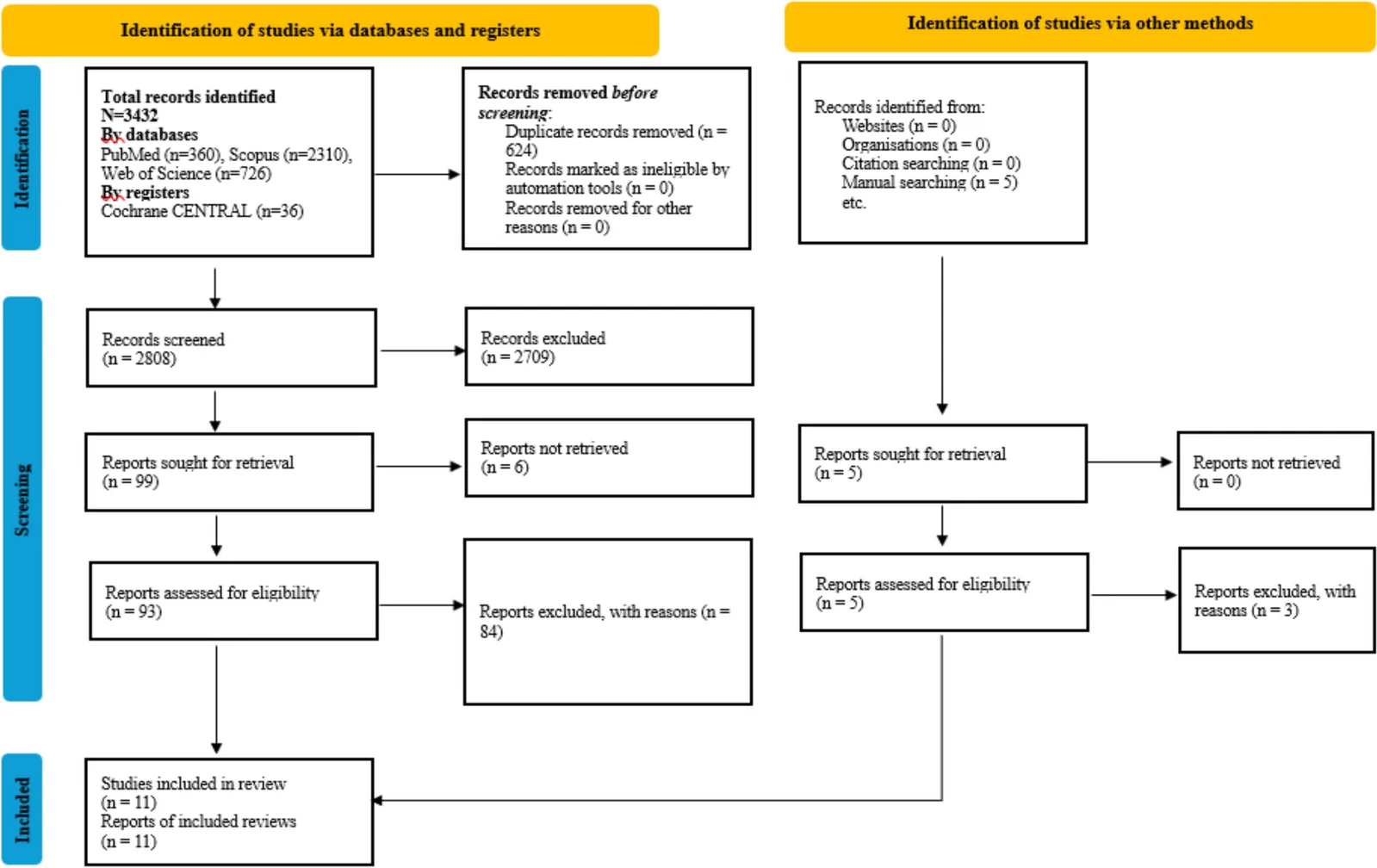
PRISMA flowchart

A total of 3432 articles were identified through database searching, with an additional 5 records through manual searching. Following the removal of 624 duplicates, the titles and abstracts of 2808 records were screened, resulting in the exclusion of 2709 articles. The full texts of 98 articles were assessed for eligibility and 87 were excluded with reasons (Appendix B), leaving 11 SRs for final inclusion in the umbrella review (Fig. 1).

### Study characteristics

From the eleven SRs that had been published between 2015 and 2025, only six reported protocol registration (Coelho et al. 2020; Jensen et al. 2021; Nassar et al. 2024; Ta et al. 2025; Triebl et al. 2024; Zainal Abidin et al. 2021). The number of primary studies included in each SR ranged from 2 to 36, rendering a total of 82 unique primary studies, with most being cross-sectional. Sample sizes varied between 174 and 2519 individuals with T1DM, and 217–2490 in healthy controls. The age of participants ranged from 2 to 19 years; yet detailed reporting of age distribution was limited across SRs. None of the included SRs reported data on diagnostic criteria or treatment characteristics of patients with T1DM. Although glycaemic control was mentioned in some SRs (Coelho et al. 2020; Ismail et al. 2015; Ta et al. 2025; Triebl et al. 2024), only one (Ta et al. 2025) provided detailed information by presenting HbA1c values reported in each primary study, without conducting a subgroup analysis.

Dental caries was mostly assessed using the DMFT/DMFS (Coelho et al. 2020; Ismail et al. 2015; Liu et al. 2021; Nassar et al. 2024; Ta et al. 2025; Triebl et al. 2024), dmft (AlJaafary et al. 2024; Coelho et al. 2020; Ismail et al. 2015; Liu et al. 2021; Nassar et al. 2024; Abduldaiem et al. 2023; Ta et al. 2025; Triebl et al. 2024), and dmfs (Coelho et al. 2020; Ismail et al. 2015) indices. Several SRs also examined other caries-specific related parameters, such as decayed teeth or surfaces, filled teeth or surfaces and missing teeth or surfaces (Coelho et al. 2020; Ismail et al. 2015; Nassar et al. 2024; Abduldaiem et al. 2023; Ta et al. 2025; Triebl et al. 2024).

Data indices on gingival inflammation and oral hygiene most frequently reported were GI (AlJaafary et al. 2024; Ismail et al. 2015; Jensen et al. 2021; Nassar et al. 2024; Rapone et al. 2020; Triebl et al. 2024; Zainal Abidin et al. 2021), followed by PI (Ismail et al. 2015; Jensen et al. 2021; Nassar et al. 2024; Rapone et al. 2020; Triebl et al. 2024; Zainal Abidin et al. 2021), Other less commonly used indices for periodontal health were calculus Index (Ismail et al. 2015; Nassar et al. 2024; Triebl et al. 2024; Zainal Abidin et al. 2021), OHI-s (Ismail et al. 2015), BoP (Ismail et al. 2015; Jensen et al. 2021; Rapone et al. 2020; Zainal Abidin et al. 2021), PPD (Jensen et al. 2021; Rapone et al. 2020; Zainal Abidin et al. 2021) and CAL (Ismail et al. 2015; Jensen et al. 2021; Rapone et al. 2020; Zainal Abidin et al. 2021).

MA was performed in eight of the included SRs (Coelho et al. 2020; Jensen et al. 2021; Liu et al. 2021; Nassar et al. 2024; Rapone et al. 2020; Ta et al. 2025; Triebl et al. 2024; Zainal Abidin et al. 2021). Of these, three MAs focused on dental caries outcomes (Coelho et al. 2020; Liu et al. 2021; Ta et al. 2025), three on periodontal outcomes (Jensen et al. 2021; Rapone et al. 2020; Zainal Abidin et al. 2021) and two assessed both caries and periodontal parameters (Nassar et al. 2024; Triebl et al. 2024).

A detailed summary of the characteristics of the included SRs is presented in Table 1.

**Table 1:** Characteristics of included SRs

Author/Year	Studies including participants < 19 years	Protocol registration/Guidelines followed/Funding	Databases searched/Date of search	Population < 19 years	Meta-analysis	Outcomes assessed
Ismail et al. 2015	32 studies (29 case–control, 3 longitudinal)	No/No/NR	PubMed/Medline, Web of Knowledge, Embase and Scopus/up to 1/2014	T1DM: 2487 Control: 2253	No	DMFT, DMFS, DFS, %DMFT > 0, DT, MT, FT, dmft, dmfs, OHI-s, PI, BoP, Calculus Index, CAL, GI, Perio index, prevalence of gingivitis / periodontal disease
Coelho et al. 2020	36 studies (36 cross-sectional)	Yes/Yes/No	Cochrane Library, Embase, PubMed and Web of Science / up to 30/4/2019	T1DM: 2519 Control: 2490	Yes	dmft, dmfs, dt, mt, ft, dfs, DMFT, DT, MT, FT, DMFS, DFS, DS, MS, FS
Rapone et al. 2020	9 studies (7 cross-sectional, 2 case–control)	No/Yes/NR	Medline / from 2004 to 2019	T1DM: 759 Control: 719	Yes	BoP, GI, PI, PPD, CAL
Jensen et al. 2021	23 studies (23 cross-sectional)	Yes/Yes/NR	PubMed and Embase / up to 2/2019	T1DM: 1803 Control: 1627	Yes	PI, GI, BoP, PPD, CAL
Liu et al. 2021	10 studies (10 cross-sectional)	No/No/No	PubMed/Medline, Cochrane Library, Web of Science, Embase and Scopus / from 1/1980 to 12/2018	T1DM: 956 Control: 865	Yes	DMFT, DMFS, dmft
Zainal Abidin et al. 2021	9 studies (9 cross-sectional)	Yes/Yes/NR	EBSCO Medline Complete, PubMed and Science Direct / from 2000 to 2019	T1DM: 632 Control: 587	Yes	PI, GI, BoP, percentage of bleeding sites, Calculus Index, CAL, PPD
Abduldaiem et al. 2023	8 studies (6 cross-sectional, 2 case–control)	No/Yes/NR	PubMed, Cochrane Library, Google Scholar and Embase / from 1/2008 to 9/2023	T1DM: 511 Control: 625	No	dmft, deft, dft, dt
Nassar et al. 2024	31 studies (26 cross-sectional, 5 case–control)	Yes/No/No	Embase, Medline/PubMed and Web of Science / up to 20/2/2023	T1DM: 2169 Control: 2188	Yes	DMFT, dmft, DMFS, MT, MS, FT, FS, DT, DS, DFT, PI, GI, Calculus Index
Triebl et al. 2024	33 studies (21 cross-sectional, 12 case–control)	Yes/Yes/Yes	PubMed, Cochrane Library and Embase / up to 30/5/2024	T1DM: 2516 Control: 2461	Yes	DMFT, dmfs, DMFS, deft, DFS, DFT, dmft, PI, Calculus Index, GI
AlJaafary et al. 2024	2 studies (2 case–control)	No/Yes/No	Google Scholar, Scopus and PubMed / from 2000 to June 2024	T1DM: 174 Control: 217	No	Prevalence of gingivitis, dmft, GI
Ta et al. 2025	14 studies (13 cross-sectional, 1 cohort)	Yes/Yes/No	PubMed, Embase, Scopus and Web of Science / up to 4/2024	T1DM: 836 Control: 969	Yes	DMFT, DMFS, dmft, DFS

*T1DM* type 1 diabetes mellitus, *NR* not reported, *DMFT* decayed missing filled permanent tooth, *DMFS* decayed missing filled permanent tooth surface, *DFT* decayed filled permanent tooth, *DFS* decayed filled permanent tooth surface, *DT* decayed permanent tooth, *MT* missing permanent tooth, *FT* filled permanent tooth, *dmft* decayed missing filled primary tooth, *dmfs* decayed missing filled primary tooth surface, *OHI-s* simplified oral hygiene index, *PI* plaque index, *BoP* bleeding on probing, *CAL* clinical attachment loss, *GI* gingival index, *dt* decayed primary tooth, *mt* missing primary tooth, *ft* filled primary tooth, *dfs* decayed filled primary tooth surface, *DS* decayed permanent tooth surface, *MS* missing permanent tooth surface, *FS* filled permanent tooth surface, *PPD* periodontal probing depth, *deft* decayed extracted filled primary tooth, *dft* decayed filled primary tooth

### Overlapping SRs

**Fig. 2:**
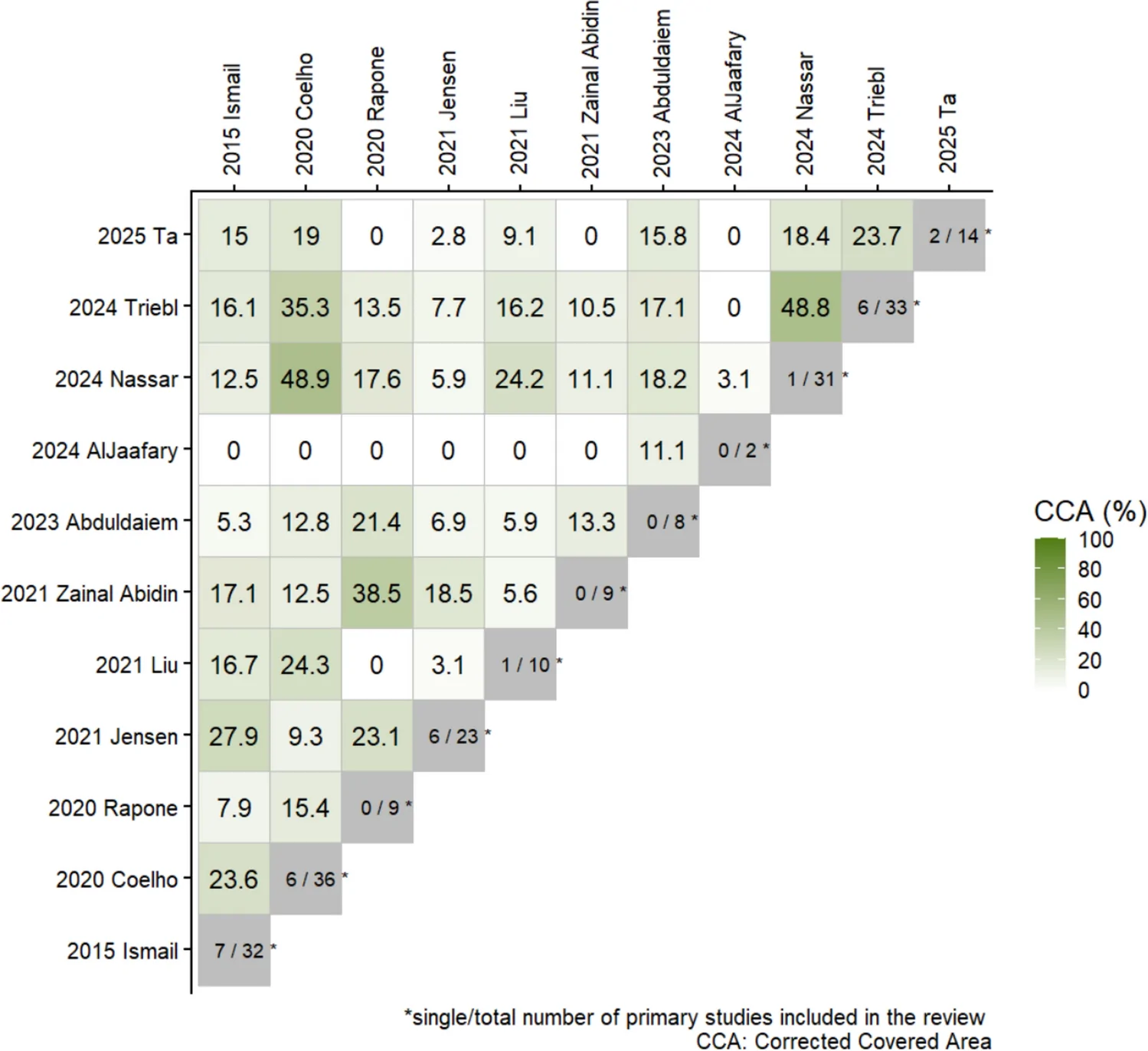
Corrected Covered Area

In total, the number of unique primary studies was 82 and the total number of included publications across the eleven SRs was 207. The CCA value was 0.152 (15.2%), indicating a very high overlap across SRs (Fig. 2). The highest overlap was observed between SRs with comparable populations, eligibility criteria and outcomes (Coelho et al. 2020; Nassar et al. 2024; Triebl et al. 2024). Accordingly, overlap was minimal in SRs with a narrow outcome focus, with some including only periodontal parameters and others restricting to dental caries experience (Liu et al. 2021; Rapone et al. 2020; Ta et al. 2025; Zainal Abidin et al. 2021).

### Quality assessment

Overall risk-of-bias was judged as low in three SRs (Coelho et al. 2020; Jensen et al. 2021; Ta et al. 2025), as high in five (AlJaafary et al. 2024; Ismail et al. 2015; Liu et al. 2021; Nassar et al. 2024; Triebl et al. 2024) and as being of unclear risk-of-bias in the remaining three (Abduldaiem et al. 2023; Rapone et al. 2020; Zainal Abidin et al. 2021) (Table 2, Fig. 2).

**Table 2:** Quality assessment of the included SRs using the ROBIS tool

Study	Quality assessment tool in SR	Review Process				Risk of Bias	Concerns
		SEC	ISS	DCSA	SF		
Ta et al. 2025	Joanna Briggs Institute appraisal tool	**–**	**–**	**–**	**–**	**–**	Minor concerns regarding data synthesis as studies of unclear risk-of-bias included in the MA
AlJaafary et al. 2024	Risk-of-bias in non-randomised studies of exposure tool	** + **	** + **	** + **	** + **	** + **	Concerns regarding pre-defined inclusion criteria, limited search databases and lack of evidence for full search strategy, study selection upon diagnosis, data reporting and synthesis, risk-of-bias assessment
Nassar et al. 2024	Not performed	** + **	** + **	** + **	** + **	** + **	Concerns regarding eligibility criteria reporting, limited search strategy and selection process, data collection and handling of missing data, quality of studies included in the MA
Triebl et al. 2024	Newcastle Ottawa Scale	**?**	**–**	**?**	** + **	** + **	Concerns regarding eligibility criteria, limited database search, data collection and reporting and data synthesis
Liu et al. 2021	Healthcare Research and Quality Agency	**?**	** + **	** + **	** + **	** + **	Concerns regarding eligibility criteria based on diagnostic criteria, selection methodology, quality assessment tool used, data collection, MA based on high risk-of-bias studies
Zainal Abidin et al. 2021	National Heart, Lung, and Blood Institute and the National Institutes of Health quality assessment tool for observational cross-sectional studies	**–**	**?**	**–**	**–**	**?**	Concerns regarding search strategy completeness, some primary studies had moderate / high risk-of-bias
Jensen et al. 2021	Joanna Briggs Institute appraisal tool	**–**	**–**	**–**	**–**	**–**	
Rapone et al. 2020	Not performed	**?**	**?**	**?**	**?**	**?**	Concerns regarding lack of risk-of-bias assessment tool for studies and methods to account for heterogeneity among included studies
Ismail et al. 2015	Not performed	**?**	**–**	** + **	**?**	** + **	Concerns regarding lack of risk-of-bias assessment tool for studies, heterogeneity and search strategy
Coelho et al. 2020	Newcastle Ottawa Scale	**–**	**–**	**–**	**–**	**–**	
Abduldaiem et al. 2023	Newcastle Ottawa Scale	**–**	**?**	**?**	**–**	**?**	Concerns regarding study selection, i.e. excluding studies of high risk and date restriction, handling of missing data

(** +)** high risk of bias; (**–)** low risk of bias; **(?)** unclear risk of bias

*SEC* study eligibility criteria, *ISS* identification and selection of studies, *DCSA* data collection and study appraisal, *SF* synthesis and findings

**Fig. 3:**
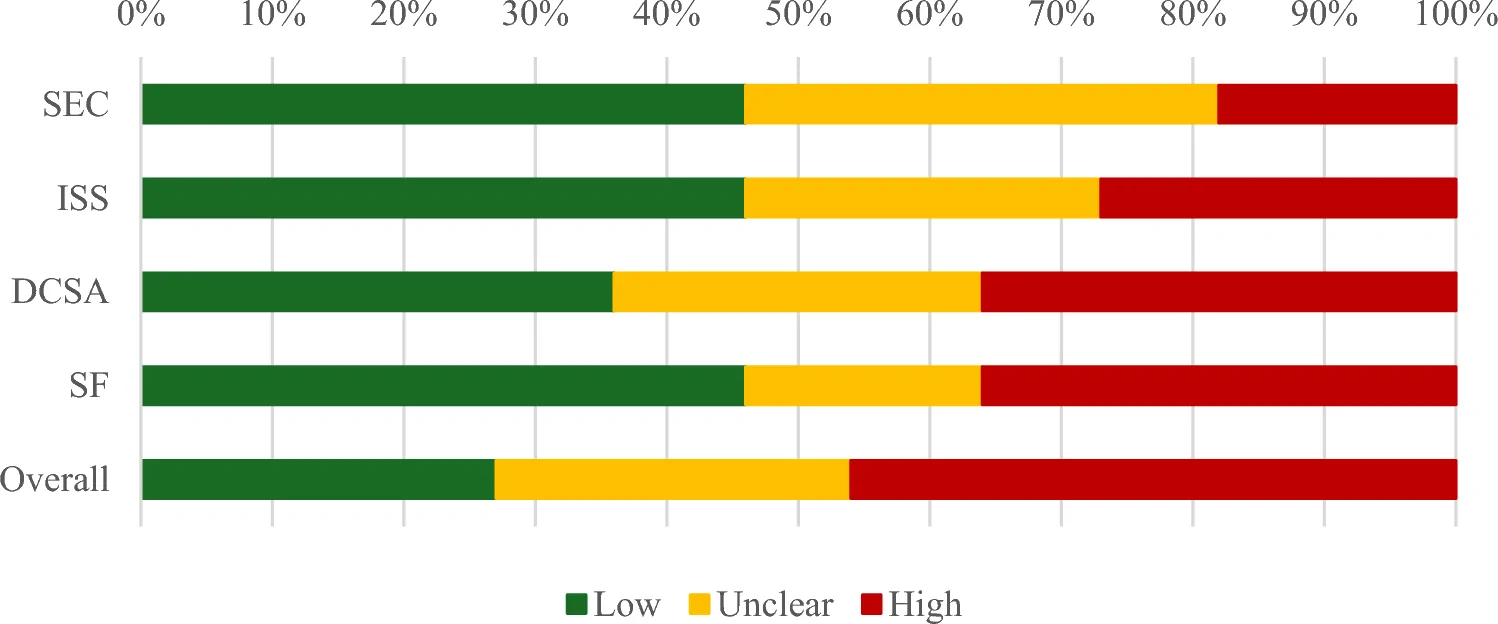
Distribution of risk of bias assessments

Concerns were noted in six SRs regarding selection process due to lack of prespecified criteria regarding diagnosis and restrictive or inadequate reporting (AlJaafary et al. 2024; Ismail et al. 2015; Liu et al. 2021; Nassar et al. 2024; Rapone et al. 2020; Triebl et al. 2024). Moreover, limitations concerning identification and selection of studies were observed in another six SRs, due to limited number of databases searched and insufficient reporting of the study selection process (AlJaafary et al. 2024; Liu et al. 2021; Nassar et al. 2024; Abduldaiem et al. 2023; Rapone et al. 2020; Zainal Abidin et al. 2021). In terms of data collection and study appraisal, seven SRs had limitations, such as absence of formal risk-of-bias assessment or inadequate description of data extraction process (AlJaafary et al. 2024; Ismail et al. 2015; Liu et al. 2021; Nassar et al. 2024; Abduldaiem et al. 2023; Rapone et al. 2020; Triebl et al. 2024). Finally, concerns related to data synthesis and findings were raised in all but three SRs, including inappropriate assessment of clinical and methodological heterogeneity, and MAs conducted without appropriate consideration of the risk-of-bias of the included primary studies (AlJaafary et al. 2024; Ismail et al. 2015; Liu et al. 2021; Nassar et al. 2024; Abduldaiem et al. 2023; Rapone et al. 2020; Triebl et al. 2024; Zainal Abidin et al. 2021). See (Fig. 3).

### Findings of the SRs

- a. Dental caries outcomes

Mean caries prevalence in permanent dentition, assessed by the percentage of teeth with DMF > 0 was 71% in T1DM and 60% in the control group. Overall calculated mean DMFT values were 3.72 for T1DM and 2.47 for controls, ranging between 0.09 to 11.49 and 0.2 to 9.56, respectively. Corresponding values for primary dentition (dmft) were lower in individuals with T1DM (mean: 2.54; range: 0.26–6.7) compared to the controls (mean: 3.3; range: 0.77–10.5).

**Fig. 4:**
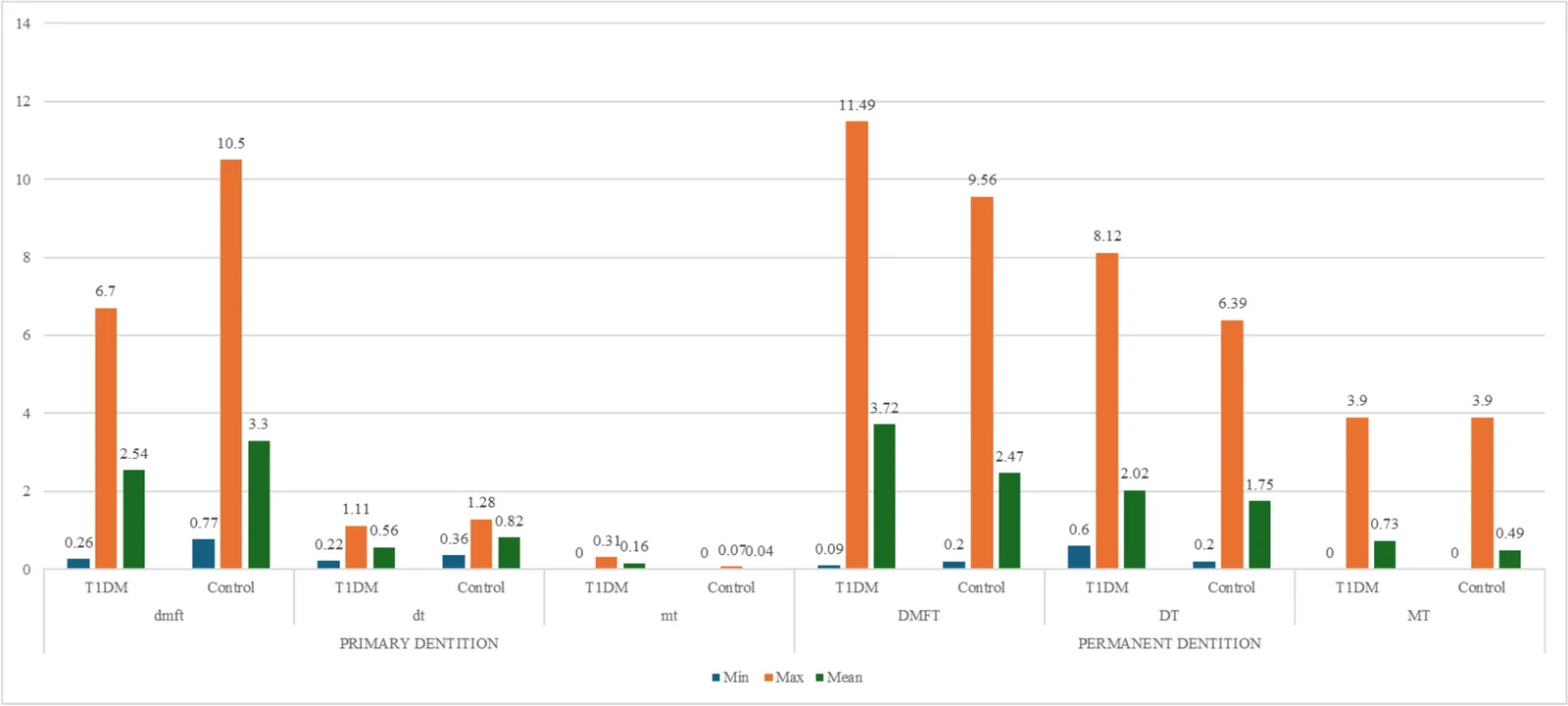
Comparison of minimum, maximum and mean dental caries values in primary and permanent dentition between T1DM and healthy controls

Calculated mean values for other caries indices (dmfs, deft, ft) were generally higher in the control group, with eight primary studies supporting a statistically significant difference, in primary dentition. In contrast, the remaining indices in permanent dentition showed a more heterogeneous pattern, with no consistent direction of effect between T1DM and the control (Table 3, Fig. 4).

- b. Periodontal outcomes

**Table 3:** Calculated mean values of dental caries in primary and permanent dentition in T1DM and control groups across the included SRs

Review	Studies	Sample		Mean values (SD) in primary dentition								Mean values (SD) in permanent dentition						
				dmft	dmfs	deft	dft	dt	mt	ft	% DMFT > 0	DMFT	DMFS	DFS	DFT	DT	MT	FT
Ismail et al. 2015	15 case–control, 1 longitudinal	T1DM	1220	1.75 (1.57)	3.61 (2.88)						100	5.4 (3.2)	15.47 (13.3)	7.47 (5.9)		4.48 (3.4)	1.38 (1.5)	1.42 (1.7)
		Control	1109	2.39 (1.58)	4.51 (3.36)						97	4.02 (2.7)	16.89 (15.6)	10.53 (8.6)		3.47 (4.1)	1.09 (1.9)	1.16 (0.4)
Coelho et al. 2020	36 cross-sectional	T1DM	2519	2.16 (1.9)				0.38 (0.2)	0.16 (0.2)	0.11 (0.1)		3.85 (2.9)	10.42 (11.9)	7.47 (5.9)		2.28 (1.8)	0.72 (1.2)	2.15 (1.9)
		Control	2490	2.85 (2.9)				0.79 (0.5)	0.04 (0.1)	0.26 (0.3)		2.76 (2.2)	10.99 (13.6)	10.53 (8.6)		1.95 (1.8)	0.56 (1.2)	1.96 (1.8)
Liu et al. 2021	10 cross-sectional	T1DM	956	2.27 (2.3)								2.87 (1.4)	9.57 (8.2)					
		Control	865	4.15 (4.1)								2.07 (1.4)	9.64 (10.3)					
Abduldaiem et al. 2023	6 cross-sectional, 2 case–control	T1DM	511	3.25 (1.8)		0.54 (0.1)	3.32	1.0										
		Control	625	5.11 (2.6)		1.08 (0.3)	3.28	0.69										
Nassar et al. 2024	26 cross-sectional, 5 case–control	T1DM	2169	2.32 (2.4)								3.82 (5.8)	5.61 (2.4)		2.11 (1.2)	1.91 (1.6)	0.74 (1.1)	1.77 (1.2)
		Control	2188	3.61 (1.4)								4.14 (5.1)	4.46 (2.3)		2.24 (1.3)	1.85 (1.3)	0.68 (0.9)	1.55 (1.4)
Triebl et al. 2024	21 cross-sectional, 12 case–control	T1DM	2516									2.94 (1.6)						
		Control	2461									2.48 (1.4)						
AlJaafary et al. 2024	1 case–control	T1DM	40	4.87 (3.97)														
		Control	40	7.17 (4.74)														
Ta et al. 2025	13 cross-sectional, 1 longitudinal	T1DM	620	3.45 (1.5)							41	4.61 (1.6)	8.20 (10.1)					
		Control	753	3.86 (3.8)							33	3.19 (1.2)	7.24 (11.4)					

*T1DM* type 1 diabetes mellitus, *DMFT* decayed missing filled permanent tooth, *DMFS* decayed missing filled permanent tooth surface, *DFT* decayed filled permanent tooth, *DFS* decayed filled permanent tooth surface, *DT* decayed permanent tooth, *MT* missing permanent tooth, *FT* filled permanent tooth, *dmft* decayed missing filled primary tooth, *dmfs* decayed missing filled primary tooth surface, *dt* decayed primary tooth, *mt* missing primary tooth, *ft* filled primary tooth, *deft* decayed extracted filled primary tooth, *dft* decayed filled primary tooth

Evidence on prevalence of gingivitis and periodontal disease was limited, with the single study from Ismail et al. (2015) reporting a significantly higher prevalence of gingivitis in children with T1DM compared to the control group (29% vs. 7%), and an insignificantly higher prevalence of periodontal disease (27% vs. 13%). Additionally, in the review by AlJaafary et al. (2024), findings from one primary study showed comparable prevalence of gingivitis amongst paediatric patients with T1DM and the controls (72.4% vs. 60%).

**Fig. 5:**
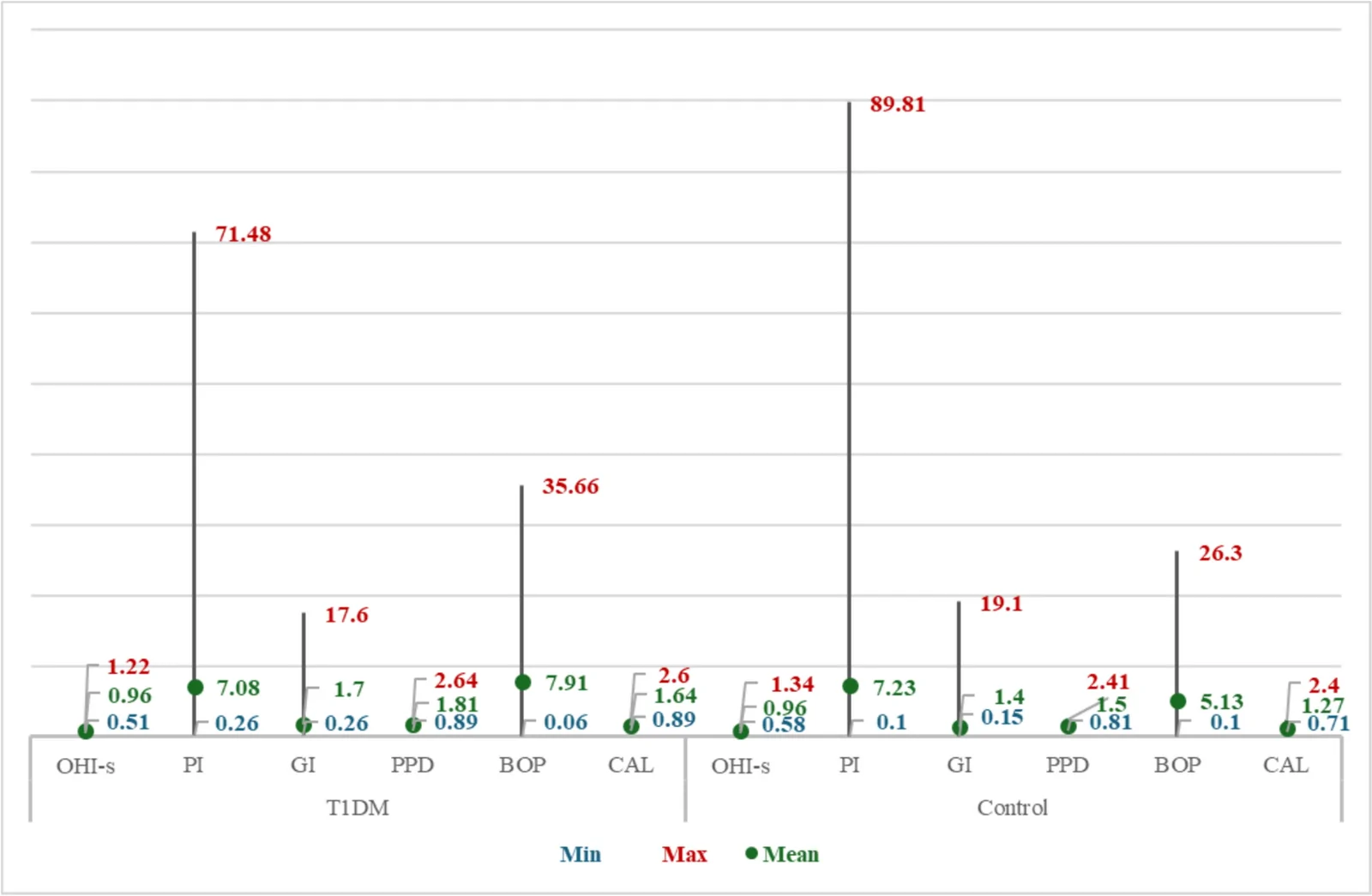
Comparison of minimum, maximum and mean periodontal values between T1DM and healthy controls

Calculated mean values for most periodontal indices showed small differences between groups (Fig. 5, Table 4). Plaque levels were generally comparable, with mean PI values of 7.08 in T1DM and 7.23 in controls, whilst GI tended to be slightly higher in T1DM (1.7 vs 1.4). Similarly, PPD and CAL showed higher mean values in T1DM compared to healthy controls (PPD: 1.81 vs 1.50; CAL: 1.64 vs 1.27), while OHI-s values overlapped between groups (0.96).

- c. Meta-analytic results

**Table 4:** Calculated mean values of periodontal indices in T1DM and control groups across the included SRs

Review	Studies	Sample (*n*)		Mean values (SD)								
				OHI-s	PI	Calculus Index	BoP	GI	PPD	CAL	% gingivitis	% healthy Periodontium
Ismail et al. 2015	24 case–control, 3 longitudinal	T1DM	1987	0.96 (0.4)	3.06 (7.8)	0.44 (0.4)	1.93 (2.8)	1.05 (0.9)			29	33
		Control	1753	0.96 (0.5)	2.43 (6.2)	0.25 (0.3)	1.37 (1.8)	0.68 (0.5)			7	43
Rapone et al. 2020	7 cross-sectional, 2 case–control	T1DM	759		19.3 (27.9)		12.09 (20.4)	3.59 (6.9)	1.49 (0.1)	1.83 (0.8)		21
		Control	719		23.96 (33.7)		8.85 (15.1)	3.62 (7.6)	1.41 (0.1)	1.33 (0.9)		1
Jensen et al. 2021	23 cross-sectional	T1DM	1803		8.68 (18.6)		10.36 (16.4)	1.79 (3.9)	1.91 (0.4)	1.61 (0.7)		
		Control	1627		10.59 (22.8)		6.7 (10.2)	1.61 (4.4)	1.68 (0.5)	1.33 (0.6)		
Zainal Abidin et al. 2021	9 cross-sectional	T1DM	632		7.15 (20.3)		0.29 (0.1)	1.55 (0.5)	1.51 (0.8)	1.85 (1.1)		
		Control	587		8.96 (26.8)		0.14 (0.1)	1.09 (0.2)	1.27 (0.6)	1.60 (1.1)		
Nassar et al. 2024	9 cross-sectional, 2 case–control	T1DM	582		15.43 (25.63)	0.38 (0.3)		1.88 (2.8)				
		Control	529		20.15 (31.7)	0.38 (0.5)		1.57 (2.2)				
Triebl et al. 2024	5 cross-sectional, 4 case–control	T1DM	608		1.40 (1.1)	0.31 (0.3)		1.2 (1.1)				
		Control	497		1.12 (0.5)	0.25 (0.3)		0.96 (0.6)				
Aljaafary et al 2024	2 case–control	T1DM	174					8.28			72.4	
		Control	217					6.88			60	

*T1DM* type 1 diabetes mellitus, *OHI-s* simplified oral hygiene index, *PI* plaque index, *BoP* bleeding on probing, *CAL* clinical attachment loss, *GI* gingival index, *PPD* periodontal probing depth

Five SRs performed MAs on dental caries outcomes (Coelho et al. 2020; Liu et al. 2021; Nassar et al. 2024; Ta et al. 2025; Triebl et al. 2024). A trend towards higher caries experience in the permanent dentition amongst children and adolescents with T1DM was observed, with increased DMFT reported in two out of three analyses (Liu et al. 2021; Ta et al. 2025; Triebl et al. 2024). No statistically significant differences were identified for DMFS (Liu et al. 2021; Nassar et al. 2024) and individual caries components, except for the MT index, which was statistically significantly higher in systemically healthy controls (Nassar et al. 2024). In the primary dentition, findings for dental caries indicated lower dmft values in T1DM in two out of three studies (Coelho et al. 2020; Liu et al. 2021; Nassar et al. 2024) (Table 5).

**Table 5:** Results from MAs on dental caries outcomes

Author/Year	Studies included	Result	Conclusions
Coelho et al. 2020	dmft (*n* = 11)	dmft: MD 0.53, 95% CI (−0.20, 1.26), *I*^2^ = 97.8%	Although dental caries experience in primary dentition is comparable between T1DM and control groups, T1DM is associated with a high dental caries risk
Liu et al. 2021	DMFT (*n* = 8) DMFS (*n* = 5) dmft (*n* = 2)	*DMFT: SMD 0.61, 95% CI (0.14, 1.08), *I*^2^ = 93% DMFS: SMD 0.09, 95% CI (−0.20, 0.38), *I*^2^ = 82% *dmft: SMD −0.43, 95% CI (−0.71, -0.15), *I*^2^ = 0%	T1DM demonstrate lower caries experience in primary dentition compared to controls, while findings for permanent dentition are inconclusive
Nassar et al. 2024	DMFS (*n* = 4) DT (*n* = 10) DS (*n* = 4) FT (*n* = 11) FS (*n* = 3) MT (*n* = 8) MS (*n* = 2) dmft (*n* = 5) DFT (*n* = 2)	DMFS: MD −8.24, 95% CI (−23.77, 7.29), *I*^2^ = 100% DT: MD 0.03, 95% CI (−0.56, 0.62), *I*^2^ = 95% DS: MD −0.26, 95% CI (−1.18, 0.66), *I*^2^ = 77% FT: MD 0.18, 95% CI (−0.09, 0.45), *I*^2^ = 95% FS: MD 0.25, 95% CI (−0.47, 0.98), *I*^2^ = 78% *MT: MD −0.03, 95% CI (−0.03, -0.03), *I*^2^ = 0% MS: MD 0.06, 95% CI (−0.11, 0.23), *I*^2^ = 0% *dmft: MD −1.08, 95% CI (−1.89, -0.26), *I*^2^ = 52% DFT: MD −0.16, 95% CI (−0.56, 0.25), *I*^2^ = 77%	Dental caries in primary dentition is lower in T1DM, while evidence on the association between T1DM and dental caries in permanent dentition remains heterogeneous
Triebl et al. 2024	DMFT (*n* = 17)	*DMFT: SMD 0.41, 95% CI (0.03, 0.78), *I*^2^ = 98%	Dental caries experience in permanent dentition is higher in T1DM
Ta et al. 2025	DMFT (*n* = 5)	DMFT: SMD 1.02, 95% CI (−0.19, 2.22), *I*^2^ = 93%	Dental caries outcomes are largely comparable between T1DM and control; differences are observed mainly in children with poorer glycaemic control

^*^statistically significant

*T1DM* type 1 diabetes mellitus, *DMFT* decayed missing filled permanent tooth, *DMFS* decayed missing filled permanent tooth surface, *DFT* decayed filled permanent tooth, *DT* decayed permanent tooth, *MT* missing permanent tooth, *FT* filled permanent tooth, *dmft* decayed missing filled primary tooth

MAs showed a clearer pattern for some periodontal parameters (Table 6), with BoP (Jensen et al. 2021; Rapone et al. 2020; Zainal Abidin et al. 2021), probing depth and attachment loss (Jensen et al. 2021; Rapone et al. 2020) being consistently higher in T1DM patients. Regarding PI and GI, findings were more variable, as some MAs reported higher PI (Jensen et al. 2021; Nassar et al. 2024) and GI (Jensen et al. 2021) values in young patients with T1DM, whereas others did not find statistically significant differences between groups (Nassar et al. 2024; Triebl et al. 2024). Non-statistically significant differences were also reported for the calculus index (Triebl et al. 2024; Zainal Abidin et al. 2021) (Table 6).

- d. Effect of glycaemic control

**Table 6:** Results from MAs on periodontal outcomes

Author/Year	Studies included	Result	Conclusions
Rapone et al. 2020	BoP (*n* = 3) PPD (*n* = 2)	*BoP: SMD 0.65, 95% CI (0.08, 1.23), *I*^2^ = 81% *PPD: SMD 0.36, 95% CI (0.16, 0.55), *I*^2^ = 0%	There is no strong evidence to support a causal effect of T1DM on periodontal inflammation
Jensen et al. 2021	PI (*n* = 20) GI (*n* = 18) BoP (*n* = 12) PPD (*n* = 8) CAL (*n* = 7)	*PI: SMD 0.45, 95% CI (0.21, 0.70), *I*^2^ = 90% *GI: SMD 0.51, 95% CI (0.28, 0.74), *I*^2^ = 86% *BoP: SMD 0.61, 95% CI (0.40, 0.82), *I*^2^ = 80% *PPD: SMD 0.55, 95% CI (0.22, 0.87), *I*^2^ = 73% *CAL: SMD 0.54, 95% CI (0.29, 0.78), *I*^2^ = 75%	Children and adolescents with T1DM are more likely to have elevated periodontal risk markers compared to their healthy counterparts
Zainal Abidin et al. 2021	BoP (*n* = 3) Calculus Index (*n* = 4)	*BoP: SMD 0.32, 95% CI (0.07, 0.58), *I*^2^ = 0% Calculus Index: SMD 0.50, 95% CI (−0.03, 1.04), *I*^2^ = 84%	Children and adolescents with T1DM exhibit poorer periodontal health than healthy controls, although findings for calculus accumulation are mixed
Nassar et al. 2024	PI (*n* = 9) GI (*n* = 8) Calculus Index (*n* = 4)	*PI: MD 0.47, 95% CI (0.06, 0.89), *I*^2^ = 97% GI: MD 0.17, 95% CI (−0.08, 0.41), *I*^2^ = 92% Calculus Index: MD 0.01, 95% CI (−0.10, 0.13), *I*^2^ = 80%	T1DM in children and adolescents is associated with increased plaque accumulation, while findings for other periodontal outcomes are heterogeneous
Triebl et al. 2024	GI (*n* = 7) Calculus Index (*n* = 3) PI (*n* = 7)	GI: MD 0.05, 95% CI (−0.01, 0.11), *I*^2^ = 44% Calculus Index: MD 0.04, 95% CI (0.00, 0.09), *I*^2^ = 0% PI: MD 0.17, 95% CI (−0.40, 0.74), *I*^2^ = 95%	No association exists between T1DM and periodontal outcomes in children and adolescents

^*^statistically significant

*T1DM* type 1 diabetes mellitus, *PI* plaque index, *BoP* bleeding on probing, *CAL* clinical attachment loss, *GI* gingival index, *PPD* periodontal probing depth

A limited number of the included SRs (Coelho et al. 2020; Ismail et al. 2015; Ta et al. 2025; Triebl et al. 2024) examined glycaemic control as a modifying factor (Table 7). An overall strong association was demonstrated between metabolic control and caries outcomes, with the poorly controlled T1DM groups showing higher caries experience and, in some cases, higher prevalence. This was further supported by one of the SRs (Triebl et al. 2024) that performed a subgroup analysis and found statistically significantly higher DMFT scores in poorly controlled subjects, whereas comparable values were found for those with well-controlled T1DM. Similarly, Ta et al. (2025) demonstrated that children with elevated HbA1c (> 7.5%) had lower proportions of caries-free children, compared to well-controlled subjects and healthy controls. However, in two of the included primary studies, no difference was observed in DMFS between patients with higher HbA1c and controls.

**Table 7:** Summary of findings on glycaemic control

Author/Year	Main findings
Ismail et al. 2015	3 year follow-up: ↑caries in children with poor metabolic control (*n* = 1); High caries risk linked to impaired metabolic control (*n* = 1)
Coelho et al. 2020	Poor glycaemic control was associated with higher caries experience (*n* = 1); ↑HbA1c levels were associated with increased caries development (*n* = 1)
Triebl et al. 2024	Poorly controlled T1DM (HbA1c > 7.5–8%) vs control: *DMFT [*n* = 5, MD 1.46 95% CI (0.57, 2.35) *I*^2^ = 92%]; Well-controlled T1DM (HbA1c < 7.5–8%) vs control: DMFT [*n* = 5, MD 0.26 95% CI (−0.50, 1.03) *I*^2^ = 91%]; Moreover, ↔ DMFS in poorly controlled T1DM (HbA1c ≥ 7.5%) vs control (*n* = 1); ↓DMFS in well-controlled T1DM (HbA1c < 7.5%) vs poorly controlled (*n* = 1)
Ta et al. 2025	↓proportion of caries-free children in T1DM with HbA1c > 7.5% compared with well-controlled T1DM and controls (*n* = 1); ↑DMFS in T1DM with HbA1c > 9% (*n* = 1); ↔ DMFS with HbA1c ≥ 7.5% vs controls (*n* = 2)

^*^statistically significant

*T1DM* type 1 diabetes mellitus, *DMFT* decayed missing filled permanent tooth, *DMFS* decayed missing filled permanent tooth surface

## Discussion

The present umbrella review synthesised evidence from eleven SR/MAs regarding oral health in children and adolescents with T1DM, in comparison to their healthy peers. Overall, dental caries findings remain inconsistent, whereas periodontal parameters tend to be worse in paediatric populations with T1DM.

In the primary dentition, most SRs reported either lower or similar caries development in paediatric populations with T1DM when compared to healthy controls (Coelho et al. 2020; Liu et al. 2021; Nassar et al. 2024). In contrast, a tendency toward increased dental caries experience in the permanent dentition was noted in T1DM; however, the other caries-related outcomes were comparable across groups and did not exhibit a trend.

As highlighted by Ismail et al. (2015), the inconsistency in findings related to dental caries may be attributed to differences in glycaemic control of children and adolescents with T1DM across the included studies. In line with this observation, the MA by Triebl et al. (2024) reported that poorly controlled T1DM children and adolescents exhibited statistically significantly higher DMFT scores compared to healthy controls, whereas no difference was found in well-controlled T1DM. Notably, children with good metabolic control are expected to have lower caries experience, since effective glycaemic control is often linked to a sucrose-restricted diet, which reduces the cariogenic potential of dental plaque (Manjushree et al. 2022). Moreover, some evidence suggests that paediatric populations with T1DM display increased counts of caries-associated microorganisms, especially *mutans streptococci* and *lactobacilli,* compared with non-T1DM controls, although these differences often did not reach statistical significance (Ta et al. 2025). However, when participants were stratified by glycaemic control, children with higher HbA1c levels experienced significant increases in cariogenic taxa, including *streptococci* group/species and *lactobacilli* genus/species, indicating that poorer glycaemic control may be linked to a more cariogenic oral microbiota (Ta et al. 2025). Therefore, analysing children with T1DM without accounting for glycaemic control may mask clinically relevant differences in caries outcomes, particularly in study samples that include patients with different levels of metabolic control and varying duration of disease.

Salivary function is an important issue in T1DM. According to recent MAs, children and adolescents with T1DM tend to exhibit significantly lower stimulated and unstimulated salivary flow rates when compared to healthy controls, with no studies reporting higher flow for the T1DM group (Liu et al. 2021; Triebl et al. 2024). In contrast, salivary acid buffering capacity does not differ significantly between paediatric patients with T1DM and healthy peers (Liu et al. 2021). This is consistent with evidence indicating that changes in salivary flow do not necessarily translate into proportional changes in acid buffering capacity (Wikner and Söder 1994). Nonetheless, alterations observed in stimulated and unstimulated saliva may be clinically relevant, since elevated glucose concentrations have been reported in both salivary conditions, promoting a more cariogenic oral environment (Twetman et al. 2002). In this context, these observations point towards a possible link between impaired salivary function and increased dental caries risk in paediatric patients with T1DM.

The findings of the present umbrella review suggest that young patients with T1DM have a less favourable periodontal tissue inflammatory profile. Some variability was observed between PI and GI across the included SRs. While both indices were often higher in children with T1DM, findings were not entirely consistent, suggesting that gingival inflammation in this population may not be solely dependent on plaque levels, but may also reflect diabetes-related alterations in host immune response and inflammatory regulation. In this regard, the consistently higher levels of gingival bleeding observed in children and adolescents with T1DM may be attributed to vascular changes related to diabetes. Microvascular complications may affect the periodontal tissues by increasing vascular fragility and making the gingiva more prone to bleeding in the presence of inflammation (Rapone et al. 2020). This could potentially explain why BoP appears to be a more sensitive indicator of periodontal involvement in children with T1DM than GI.

Another association was observed between T1DM and periodontal tissue breakdown parameters (PPD, CAL) in a paediatric population (Jensen et al. 2021; Rapone et al. 2020). This finding indicates that young patients with T1DM not only experience gingival inflammation but also show early structural damage of the periodontal tissues. Although alterations in oral microbiota have been reported in children with T1DM, these appear to be inconsistent and influenced by glycaemic control and periodontal health status (Vlachou et al. 2024). Taken together, these findings support the concept that periodontal destruction in T1DM is primarily caused by host-mediated inflammatory responses rather than microbial shifts unique to diabetes (Lalla and Papapanou 2011). In this context, changes in the innate immune function due to diabetes affect polymorphonuclear leukocytes (PMNs). In T1DM, PMNs are dysfunctional, exhibiting decreased chemotaxis, phagocytosis and resolution of inflammation. This results in their prolonged presence within periodontal tissues and continued secretion of reactive oxygen species (ROS) and enzymes that facilitate periodontal destruction (Preshaw et al. 2012). Moreover, chronic hyperglycaemia promotes the formation and accumulation of advanced glycation end products (AGEs) in the periodontal tissues. Interaction of AGEs with their receptor (RAGE) further intensifies the inflammatory response, by producing pro-inflammatory mediators, including TNF-α, IL-6 and IL-1β, which contribute to tissue destruction (Lalla et al. 2001; Preshaw et al. 2012).

However, data on the prevalence of periodontal disease were limited and inconclusive; thus, it seems that advanced periodontal breakdown is uncommon in childhood or adolescence (Ismail et al. 2015; Chapple et al. 2026). It is also important to mention that none of the included SRs evaluated periodontal outcomes according to glycaemic control, although it is an acknowledged risk factor for periodontal disease (Bissett et al. 2015).

## Strengths and limitations

This umbrella review has several methodological strengths. The review protocol was prospectively registered in PROSPERO, and reporting followed the PRIOR statement, thereby enhancing transparency and reproducibility (Gates et al. 2022). Eligibility criteria were also clear and prespecified, allowing for consistent selection of paediatric populations and relevant oral health outcomes. By synthesising evidence according to oral health outcomes rather than SRs, comparisons across periodontal and dental caries indices were facilitated. In addition, to address the inherent risk of double counting in umbrella reviews, overlap between SRs was formally assessed using CCA (Pieper et al. 2014). In parallel, the evaluation of methodological quality using the ROBIS tool allowed for consideration of potential bias when interpreting the results (Whiting et al. 2016).

Nonetheless, several limitations should be acknowledged. A common issue was the absence of clear diagnostic definitions for T1DM, as some SRs included mixed populations with DM (T1DM, T2DM, unspecified type) or failed to report diabetes classification clearly (AlJaafary et al. 2024; Coelho et al. 2020; Zainal Abidin et al. 2021). This may have influenced the internal validity of the included SRs, even though mixed-population syntheses were not reported in the present umbrella review. In certain cases, primary studies were included in MAs despite not meeting all eligibility criteria, whilst discrepancies existed between the number of studies reported in PRISMA flow diagrams and those eventually included in the synthesis. Moreover, certain studies that met the inclusion criteria appeared in SRs without being clearly integrated into the synthesis, which could affect the reliability of the pooled estimate (Nassar et al. 2024). It should also be mentioned that not all included SRs performed a formal risk-of-bias assessment, which should be taken into account when interpreting the findings. Furthermore, the restriction to English language publications represents another limitation, since relevant evidence published in other languages may not have been captured.

Finally, several key clinical modifiers were not well-described or adjusted for across the SRs, despite their potential to have an impact on both dental caries and periodontal outcomes. Specifically, glycaemic control was not discussed commonly as part of the interpretation of the findings, nor was it incorporated regularly into subgroup analyses. Information regarding diabetes treatment status, including insulin regimens or other therapeutic approaches, was not provided, restricting the ability to assess whether this factor may have influenced oral health outcomes. Thus, future SRs would benefit from clearer population definitions, more consistent outcome assessments, consideration of treatment and disease duration, and overall, more rigorous and transparent reporting.

## Conclusions

- With regard to dental caries, findings were heterogeneous and differed between dentitions. In the primary dentition, caries experience in children and adolescents with T1DM was generally comparable to or lower than that of healthy peers. As for the permanent dentition, dental caries outcomes were mixed, with a tendency to higher DMFT values in T1DM, which appeared to be influenced by worse glycaemic control.
- In contrast, periodontal parameters showed a more consistent pattern, with children with T1DM experiencing higher gingival inflammation and periodontal tissue destruction compared to healthy controls.
- These findings highlight the importance of early preventive oral care, regular follow-ups and close collaboration between dental and medical professionals in the long-term management of children and adolescents with T1DM.

## Supplementary Information

Below is the link to the electronic supplementary material.

Supplementary file1 (DOCX 16 KB)

Supplementary file2 (DOCX 15 KB)

## Data Availability

The data supporting the findings of this study are available from the corresponding author upon reasonable request.
