# Supplementary material for: Dental caries and periodontal status in children and adolescents with type 1 diabetes mellitus: an umbrella review
Source: Eur Arch Paediatr Dent. 2026 Jun 10;27(5):1335–51. doi: 10.1007/s40368-026-01224-9 (PMC13593804; doi:10.1007/s40368-026-01224-9)
Supplement: Supplementary file 1 [file 40368_2026_1224_MOESM1_ESM.docx]

# **APPENDIX A. Search strategy**

| **Database (assessed on 30/08/2025)** | **Search query** | **Results** |
| --- | --- | --- |
| PubMed | ("Diabetes Mellitus"[MeSH] OR "type 1 diabetes" OR "type I diabetes" OR T1DM OR diabetic OR IDDM OR "juvenile diabetes" OR "insulin dependent") AND ("Dental Caries"[MeSH Terms] OR "DMF Index"[MeSH Terms] OR caries OR decay OR dmft OR "caries index" OR “Periodontal Diseases"[MeSH Terms] OR "Dental Plaque Index"[MeSH Terms] OR "Oral Hygiene Index"[MeSH Terms] OR "Periodontal Index"[MeSH Terms] OR periodontitis OR periodont* OR gingivitis OR gingiv* OR "plaque index" OR "calculus index" OR "gingival index" OR "clinical attachment loss" OR "oral hygiene" OR "bleeding on probing"). The “*Systematic* *Review*” and “*Meta-Analysis*” filters were also applied. | 360 |
| Cochrane Library | #1 "Diabetes Mellitus"[MeSH]  #2 "type 1 diabetes"  #3 "type I diabetes"  #4 "Diabetes Mellitus, Type 1"[MeSH]  #5 diabetic  #6 IDDM  #7 T1DM  #8 "juvenile diabetes"  #9 "insulin dependent"  #10 #1 OR #2 OR #3 OR #4 OR #5 OR #6 OR #7 OR #8  #11 "Dental Caries"[MeSH]  #12 "DMF Index"[MeSH]  #13 caries  #14 decay  #15 dmft  #16 "caries index"  #17 "Periodontal Diseases"[MeSH]  #18 "Dental Plaque Index"[MeSH]  #19 "Oral Hygiene Index"[MeSH]  #20 "Periodontal Index"[MeSH]  #21 periodontitis  #22 periodont*  #23 gingivitis  #24 gingiv*  #25 "plaque index"  #26 "calculus index"  #27 "gingival index"  #28 "clinical attachment loss"  #29 "oral hygiene"  #30 "bleeding on probing"  #31 #11 OR #12 OR #13 OR #14 OR #15 OR #16 OR #17 OR #18 OR #19 OR #20 OR #21 OR #22 OR #23 OR #24 OR #25 OR #26 OR #27 OR #28 OR #29 OR #30  #32 #10 AND #31  *Trials* were excluded. | 36 |
| Scopus | TITLE-ABS-KEY ( "diabetes mellitus" OR "type 1 diabetes" OR "type I diabetes" OR T1DM OR diabetic OR IDDM OR "juvenile diabetes" OR "insulin dependent" ) AND TITLE-ABS-KEY ( "dental caries" OR "DMF index" OR caries OR decay OR dmft OR "caries index" OR "periodontal disease" OR "dental plaque index" OR "oral hygiene index" OR "periodontal index" OR periodontitis OR periodont* OR gingivitis OR gingiv* OR "plaque index" OR "calculus index" OR "gingival index" OR "clinical attachment loss" OR "oral hygiene" OR "bleeding on probing" ) AND ( LIMIT-TO ( DOCTYPE , "re" ))  Limited to *Review* | 2310 |
| Web of Science | TS=("diabetes mellitus" OR "type 1 diabetes" OR "type I diabetes" OR T1DM OR diabetic OR IDDM OR "juvenile diabetes" OR "insulin dependent") AND TS=("dental caries" OR "DMF index" OR caries OR decay OR dmft OR "caries index" OR "periodontal disease" OR "dental plaque index" OR "oral hygiene index" OR "periodontal index" OR periodontitis OR periodont* OR gingivitis OR gingiv* OR "plaque index" OR "calculus index" OR "gingival index" OR "clinical attachment loss" OR "oral hygiene" OR "bleeding on probing")  Limited to *Review Article* | 726 |
