# Supplementary material for: Dental caries and periodontal status in children and adolescents with type 1 diabetes mellitus: an umbrella review
Source: Eur Arch Paediatr Dent. 2026 Jun 10;27(5):1335–51. doi: 10.1007/s40368-026-01224-9 (PMC13593804; doi:10.1007/s40368-026-01224-9)
Supplement: Supplementary file 2 [file 40368_2026_1224_MOESM2_ESM.docx]

# **APPENDIX B. Excluded studies and reasons for exclusion**

| **Reason for exclusion** | **Study** |
| --- | --- |
| Wrong population | Alsharari et al. 2025; Weijdijk et al. 2023; Alghazaly et al. 2024; Mahajan et al. 2021; Mauri-Obradors et al. 2017; Pecci-Lloret et al. 2022; Zheng et al. 2021; Ziukaite et al. 2017; Maia et al. 2023; de Lima et al. 2020; Mirzaei et al. 2021; Stöhr et al. 2021; Borgnakke et al. 2013; Shah et al. 2024 |
| Wrong exposure | Zhou et al. 2025 |
| Wrong type of study | Bimstein et al. 2019; Buduneli 2022; Yang et al. 2024; Preshaw and Bissett 2013; He et al. 2022; Sabella et al. 2021; Oh et al. 2002; Iughetti et al. 1999; Davidopoulou et al. 2022; Meyle and Gonzáles 2001; Daković et al. 2015; Dahms 1991; Manfredi et al. 2004; Negrini et al. 2021; Ahmad et al. 2019; Ponte et al. 2001; Gürsoy et al. 2024; Ryan et al. 2003; Prato and Shetty 2022; Chandran et al. 2022; Sahu and Patnaik 2019; Sampaio et al. 2011; Rønningen and Enersen 2012; Petkova 2008; Jin et al. 2003; Lalla and Lamster 2012; Taylor and Borgnakke 2008; Nagasawa et al. 2010; Fu et al. 2025; D'Aiuto et al. 2004; Negrato et al. 2013; Pradhan and Goel 2011; Murrah 1985; Torino et al. 1990; Hayes et al. 2012; Al-Maskari et al. 2011; Lakschevitz et al. 2011; Kumar et al. 2014; Aravindha Babu et al. 2014; Liccardo et al. 2019; Minty et al. 2019; Chung et al. 2024; Parihar et al. 2024; McKenna 2006; Katariya 2015; Wolff 2014; Firat and Dağ 2009; Mirza et al. 2010; Ranbhise et al. 2025; Álamo et al. 2011; Laddha et al. 2019; Novotna et al. 2015 |
| No separate paediatric data | Alwithanani 2023; Dicembrini et al. 2020; Khader et al. 2006 |
| Wrong comparator | Costa et al. 2023; Di Spirito et al. 2025; Boy et al. 2024; Wang et al. 2019 |
| Wrong outcome | D’Agostino et al. 2024; Hatipoğlu et al. 2022; Nguyen et al. 2020; Nascimento et al. 2018; Genco and Genco 2013; Anil et al. 2025; Yousief et al. 2023 |
| Non-English article | Hilt and Filipińaska-Skapska 2007; Cabała et al. 2006; Burzyńska and Mierzwińska-Nastalska 2011; Dai and Ding 2023; Na and Chung 2016; Katagiri and Izumi 2012 |
